# Standard operating procedures for standardized mass rearing of the dengue and chikungunya vectors *Aedes aegypti* and *Aedes albopictus* (Diptera: Culicidae) - II - Egg storage and hatching

**DOI:** 10.1186/s13071-015-0951-x

**Published:** 2015-06-26

**Authors:** Min-Lin Zheng, Dong-Jing Zhang, David D. Damiens, Rosemary Susan Lees, Jeremie R.L. Gilles

**Affiliations:** Insect Pest Control Laboratory, Joint FAO/IAEA Division of Nuclear Techniques in Food and Agriculture, International Atomic Energy Agency, Vienna, Austria; Beneficial Insects Institute, Fujian Agriculture and Forestry University, Fuzhou, Fujian Province China

**Keywords:** Bacterial broth, Boiled water, Hatch rate, Egg storage, Mass rearing, Mosquito production, Aedes, Dengue, Chikungunya

## Abstract

**Background:**

Management of large quantities of eggs will be a crucial aspect of the efficient and sustainable mass production of mosquitoes for programmes with a Sterile Insect Technique component. The efficiency of different hatching media and effectiveness of long term storage methods are presented here.

**Methods:**

The effect on hatch rate of storage duration and three hatching media was analysed: deionized water, boiled deionized water and a bacterial broth, using Two-way ANOVA and Post hoc Tukey tests, and the Pearson correlation coefficient was used to find the effect on the proportion of collapsed eggs. Two long term storage methods were also tested: conventional storage (egg paper strips stored in zip lock bags within a sealed plastic box), and water storage (egg papers in a covered plastic cup with deionized water). Regression analyses were used to find the effect of water storage and storage duration on hatch rate.

**Results:**

Both species hatched most efficiently in bacterial broth. Few eggs hatched in deionized water, and pre-boiling the water increased the hatch rate of *Ae. aegypti*, but not *Ae. albopictus*. A hatch rate greater than 80 % was obtained after 10 weeks of conventional storage in *Ae. aegypti* and 11 weeks in *Ae. albopictus*. After this period, hatching decreased dramatically; no eggs hatched after 24 weeks. Storing eggs in water produced an 85 % hatch rate after 5 months in both species. A small but significant proportion of eggs hatched in the water, probably due to combined effects of natural deoxygenation of the water over time and the natural instalment hatching typical of the species.

**Conclusions:**

The demonstrated efficiency of the bacterial broth hatching medium for both *Ae. albopictus* and *Ae. aegypti* facilitates mass production of these two important vector species in the same facility, with use of a common hatching medium reducing cost and operational complexity. Similarly the increased hatch rate of eggs stored in water would allow greater flexibility of egg management in a large programme over the medium term, particularly if oxygenation of the water by bubbling oxygen through the storage tray could be applied to prevent hatching during storage.

## Background

Researchers are seeking effective and environmentally friendly methods to control arthropod-borne diseases, as the effectiveness of traditional chemical insecticides is weakened by increasing insecticide resistance, and due to concerns about negative side effects on non-target species and environmental pollution [1, 2]. Methods such as the Sterile Insect Technique (SIT) using irradiated males [3–5], the Incompatible Insect Technique (IIT) using *Wolbachia*-infected males [6–8] or use of genetically modified mosquito strains such as those carrying RIDL constructs [9–11] are potential tools for inclusion in area-wide integrated pest management (AW-IPM) programmes aiming to suppress natural mosquito populations.

All of these potential vector control techniques are based on inducing sterility in the natural population through frequent releases of large numbers of treated male mosquitos. Thus there is a need for a sustainable and effective mass production system for mosquitoes. The Insect Pest Control Laboratory (IPCL) of the Joint FAO/IAEA Division of Nuclear Techniques in Food and Agriculture, Seibersdorf (Austria) has been developing the SIT package for *Aedes aegypti* (Linnaeus) and *Ae. albopictus* (Skuse), principal vectors of dengue and chikungunya. Adult mass-rearing cages [12] and a larval mass-rearing unit [13], have been developed for *Aedes* species. Management of the large quantity of eggs produced from the mass-rearing cage is bound to be a crucial factor in maintaining the high efficiency and sustainability of a mosquito mass-rearing facility. Key aspects of egg management will be the ability to store eggs whilst maintaining high viability over time, and then to achieve a high rate of hatching when required.

Hatching is induced in *Aedes* eggs by depletion of the dissolved oxygen in the surrounding water [14, 15]; in nature, this is caused by biotic activities in the inundated egg habitat. Historically, the most commonly used protocols to remove oxygen from hatching water were boiling the water [15], bubbling nitrogen gas through the water [16], adding ascorbic acid [17, 18], or adding yeast. Yeast has been extensively used, alone or associated with larval food for *Ae. aegypti* [19–21] and *Ae. triseriatus* [22].

In mass rearing facilities, management of a large number of eggs will be critical since the ability to store *Aedes* eggs to allow the simultaneous hatching of millions of eggs is essential, for example in preparing material for release. The purpose of this paper is to describe the optimization of storage and hatching methods for *Ae. albopictus* and *Ae. aegypti* eggs. The current method for hatching *Ae. albopictus* eggs at the IPCL, submersion in a sealed glass jar containing a suspension of Nutrient Broth as used by Bellini et al. [23] but with the addition of yeast, was compared to submersion in water that had been boiled and allowed to cool, a cheaper method of deoxygenation. The impact of storage duration on egg quality using a conventional methodology was also compared to the impact of storage in deionized water.

## Methods

### Maintenance of experimental colonies

The *Ae. aegypti* and *Ae. albopictus* laboratory colonies used in all experiments originated from Juazeiro, Brazil and Rimini, Italy, respectively. About 4000–5000 adults each were kept in 60 × 60 × 60cm cages (Bioquip, Rancho Dominguez, Ca.) in a climate controlled room at a constant 25 ± 1 °C air temperature, 70 ± 5 % RH, and a photoperiod of 12:12(L:D)h. Blood meals were offered to females three times per week and a 10 % sugar solution was available throughout. Larvae were reared in plastic trays (30 × 40 × 8 cm) each containing 3000 larvae in 1 l of deionized water at a constant air temperature of 27 ± 1 °C and a photoperiod of 12:12 (L:D) h, and fed IAEA 2 larval food [24] according to the feeding regime described by Balestrino et al. [12].

### Egg collection, drying and storage

Females were provided with cylindrical containers (diameter 11.4 cm, height 9.7 cm, BioQuip, Rancho Dominguez, Ca.), containing deionized water and lined with crêpe paper (Sartorius Stedim Biotech GmbH, Göttingen, Germany) for oviposition. Egg-papers were removed every day, gently rinsed with deionized water using a plastic washing bottle to remove the dead mosquitoes, and transferred to a covered plastic tray (30 × 40 × 8 cm) for gentle drying at 27 ± 1 °C and 70 % RH for 24-48h. Egg papers were then put in plastic zip lock bags and kept in a sealed black plastic box for maturation and storage in the larval rearing room mentioned above.

### Effect of three hatching media on hatch rate of eggs

Three hatching media were tested: deionized water (hereafter called ‘DW’), boiled deionized water (‘BDW’) and bacterial broth (‘BB’, 0.7 l of deionized water, 0.25g of CM0001 Nutrient Broth (Oxoid, Hampshire, England) and 0.05g of yeast). Eggs which had been stored for either 7 or 15 days were used in this experiment. For each species and each storage duration, nine small pieces of egg papers (three per hatching medium) each containing around 300 eggs were cut from the same egg paper and completely submerged in the hatching medium in covered 100ml plastic cups in the larval rearing room mentioned above. Hatch rate was calculated after 72 h by dividing the number of eggs observed with an opercula and considered to be hatched by the total number of eggs present on the paper. Hatch rate in the DW and BDW treatments was very low after the 72h, so the egg papers were removed from their original treatment and placed in bacterial broth (BB) for a further 72h before hatch rate was rescored.

### The effect of storage duration on hatch rate

After eggs had been collected and prepared for storage as described above, the egg-paper was cut into small strips, each carrying about two to three hundred eggs. Two egg storage methods were tested. In the ‘conventional storage’ treatment egg paper strips were put in plastic zip lock bags and kept in a sealed black plastic box. Every week, for the first 10 weeks, three egg paper strips were taken out randomly from the box. Photos were taken to estimate the number of eggs which had completely collapsed. Papers were then submerged in 100ml plastic cups filled with BB, and the hatch rate of each strip was calculated. Papers were sampled for hatch rate calculation every week for the first 10 weeks of the experiment, and every month thereafter. A second, novel method, known as ‘water storage’ was also tested alongside conventional storage. After maturation, each egg paper strip was isolated in a small plastic cup filled with 100ml deionized water, covered and stored on the shelf of the larval rearing room described above. Every month for 5 months, three strips were removed from their cups. Eggs were counted and the number of eggs that hatched in the water during storage estimated by observation of a sample of eggs to calculate the “storage hatch rate”. Egg papers were then submerged into individual cups each filled with 100ml BB hatching solution. The hatch rate of eggs on each strip was calculated after 48h, termed the “final hatch rate”.

### Statistical analysis

The effect of hatching medium and storage duration on the hatch rate, and interaction between hatching medium and storage duration, were analysed with Two-way ANalysis Of Variance (ANOVA). The Post hoc Tukey test was used to test for significant differences between hatch rate according to the hatching medium and/or the storage duration.

To study the relationship between the storage period and proportion of collapsed eggs, the Pearson correlation coefficient were calculated and tested.

To analyze the change in hatch rate of eggs stored in water, regression analyses were used. First the relationship between hatch rate and duration of storage was tested for linearity, and then slopes were tested for significant difference from 0, which would indicate an effect of storage duration.

Graphics were produced and statistical analyses performed using Microsoft Excel 2003 (Microsoft, WA, USA; 1985–2003) and Minitab release 13.32 (Minitab Inc., Pennsylvania).

### Ethics statement

The blood used for routine blood-feeding was collected in Vienna, Austria during routine slaughtering of pigs or bovines in a national authorized abattoir at the highest possible standards strictly following EU laws and regulations.

## Results

### Effect of three hatching medium on hatch rate of eggs

Hatch rates (± SE) of *Ae. aegypti* and *Ae. albopictus* eggs varied significantly according to hatching media (F = 3252.85, DF = 2, P < 0.001; F = 12000, DF = 2, P < 0.001 for *Ae. aegypti* and *Ae. albopictus*, respectively) and storage duration (F = 80.46, DF = 1, P < 0.001; F = 9.41, DF = 1, *P* < 0.01 for *Ae. aegypti* and *Ae. albopictus*, respectively) (Table 1). A significant interaction between hatching medium and storage duration was also observed (F = 50.05, DF = 2, P < 0.001; F = 52.86, DF = 2, P < 0.001 for *Ae. aegypti* and *Ae. albopictus*, respectively), meaning that the effect of one variable was significantly different for each value of the other variable. Tukey post-tests indicate that there were significant differences in hatch rate between hatching media for both storage duration, and between storage duration for all hatching media.

**Table 1:** Hatch rate (mean ± SE) of *Aedes aegypti* and *Ae. albopictus* eggs in three hatching media (BB: bacterial broth, DW: deionized water, BDW: boiled deionized water during 72h after egg immersion

	Egg age (d)	Hatch rate (%)		
		BB	DW	BDW
*Ae. aegypti*	7	95.32 ± 1.14	0.42 ± 0.10	74.31 ± 1.40
	15	91.87 ± 1.23	0.10 ± 0.10	52.15 ± 1.88
*Ae. albopictus*	7	89.66 ± 0.42	1.68 ± 0.20	9.43 ± 1.03
	15	90.31 ± 0.88	0.39 ± 0.27	5.25 ± 0.60

Eggs from the DW and BDW treatments were afterwards submerged in BB to determine whether the low hatch rate was really due to the hatching medium and not due to the quality of the egg batches. Egg hatch rate after this initial period of submersion in DW or BDW was similar to the hatch rate of eggs submerged directly in BB (Table 2). Moreover there was no effect of the initial treatment (F = 0.43, DF = 1, P =0.53; F = 0.52, DF = 1, P = 0.49 for *Ae. aegypti* and *Ae. albopictus*, respectively) nor the storage duration (F = 2.33, DF = 1, P = 0.165; F = 1.82, DF = 1, P = 0.21 for *Ae. aegypti* and *Ae. albopictus*, respectively) on the final hatch rate. There was no significant interaction between previous treatment and storage duration (F = 0.22, DF = 2, P =0.65; F = 0.12, DF = 1, P =0.74 for *Ae. aegypti* and *Ae. albopictus*, respectively).

**Table 2:** Hatch rate (mean ± SE) of *Aedes albopictus* and *Aedes aegypti* eggs after immersion in Bacterial Broth (BB) after an initial immersion in deionized water (DW) and boiled deionized water (BDW)

	Storage duration(d)	Hatch rate			ANOVA
		BB	DW	BDW	DF = 2,6
*Ae.aegypti*	7	**95.32 ± 1.14**	94.55 ± 0.75	94.74 ± 1.09	F = 0.16, P = 0.86
	15	**91.87 ± 1.23**	92.61 ± 0.94	93.70 ± 1.08	F = 1.92, P = 0.23
*Ae.albopictus*	7	**89.66 ± 0.42**	90.60 ± 0.8	91.60 ± 0.8	F = 0.71, P = 0.53
	15	**90.31 ± 0.88**	92.13 ± 0.95	92.48 ± 1.06	F = 1.45, P = 0.31

ANOVA results indicate a significant difference between the control hatch rate from BB and the hatch rate after the two treatments BW and BDW first and then BB. The hatch rate indicates the total hatch rate (from the initial experiment (see Table 1) and from the test of DW to BB and BDW to BB. The hatch rate was calculated from the number of eggs that hatched in the first treatment plus the number of eggs that hatched during the second treatment in BB. Text in bold indicates that data is the report of hatch rate from BB treatment reported in Table 1, reproduced for comparison

### The effect of storage duration on hatch rate

#### Conventional storage

**Fig. 1:**
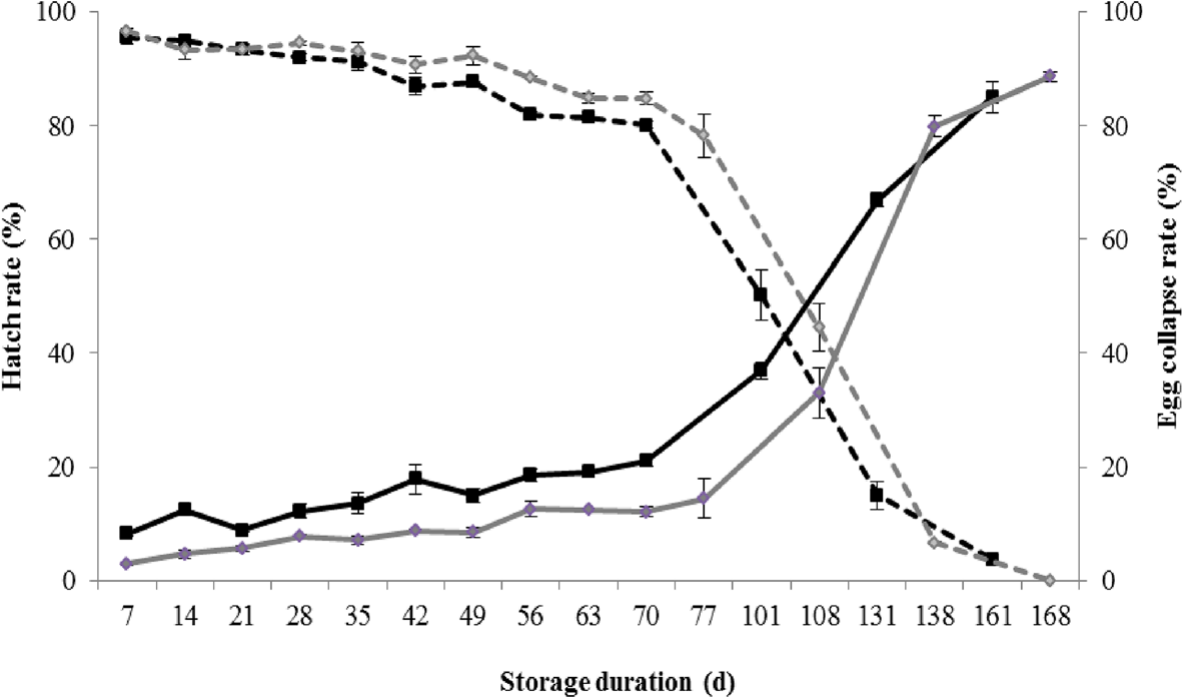
Effect of storage duration on egg hatch rate (Mean ± SE, dashed line) and on percentage of collapsed eggs (Mean ± SE, solid line) for *Aedes aegypti* (in black) and *Ae. albopictus* (in grey) in conventional storage condition

**Fig. 2:**
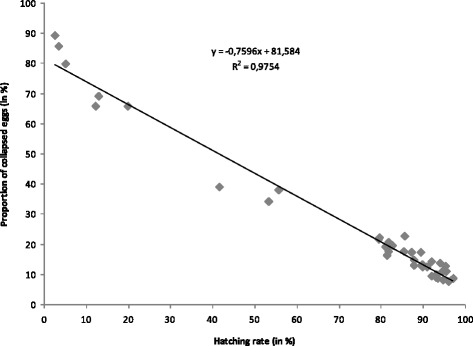
Relationship between egg hatch rate and percentage of collapsed eggs, observed on egg laying paper

Hatch rate was greater than 80 % after 10 weeks of storage for *Ae. aegypti* and after 11 weeks for *Ae. albopictus* (Fig. 1). After this period, the hatch rate of eggs decreased dramatically to 0 % after 24 weeks. The increase in the proportion of the collapsed eggs with storage time appeared to be symmetrical: few eggs collapsed after a storage of less than 10 weeks. From 10 weeks to 24 weeks of storage, the proportion of collapsed eggs increased progressively to 90 %. A significant linear relationship existed between the hatch rate and the proportion of collapsed eggs (S = 3.678, *P* < 0.0001; *r* = 0.987) (Fig. 2).

#### Water storage

Regardless of species or whether storage or final hatch rates were compared, the relationships between hatch rate and storage duration were significant (Table 3). There was a significant effect of storage duration on storage and on final hatch rate for *Ae. aegypti* since the slope of the linear regression was significantly different from zero (ANOVA, F_1,13_ = 27.7, *P* < 0.001; ANOVA, F_1,13_ = 8.9, *P* < 0.05 for storage and final hatch rates, respectively). For *Ae. albopictus*, the hatch rate after storage increased with the duration of storage (ANOVA, F_1,13_ = 11.9, *P* < 0.005) while the slope of the final hatch rate regression did not show a significant difference from zero (ANOVA, F_1,13_ = 3.6, *P* = 0.08). If hatch rates were compared between 23 weeks of conventional storage and 22 weeks (5 months) of water storage, differences were extremely significant for both species: 0.1 ± 0.1 versus 84.9 ± 9.8 %, respectively, for *Ae. albopictus *(*T* test, t = 14.9589, df = 4, P < 0.0001) and 3.7 ± 1.2 versus 84.3 ± 4.0 %, respectively, for *Ae. aegypti* (*T* test, t = 33.6313, df = 4, P < 0.0001).

**Table 3:** Hatch rate according to the duration of storage (mean ± SD) for *Aedes albopictus* and *Aedes aegypti* eggs stored submerged in deionized water

	*Aedes aegypti*		*Aedes albopictus*	
Storage duration	Storage HR in %	Final HR in %	Storage HR in %	Final HR in %
1 month	5.5 ± 0.6	95.6 ± 0.2	0.1 ± 0.2	91.3 ± 1.8
2 month	10.9 ± 1.3	95.5 ± 1.7	0.3 ± 0.3	90.0 ± 1.9
3 month	8.8 ± 2.3	95.2 ± 0.6	1.0 ± 1.0	91.6 ± 3.1
4 month	10.8 ± 1.8	84.9 ± 3.44	7.6 ± 4.5	87.7 ± 0.6
5 month	11.22 ± 1.3	84.2 ± 0.1	7.9 ± 6.9	84.9 ± 9.8
R and P	0.64/**	−0.82 /***	0.69/**	−0.46 /*

Storage HR indicates the hatch rate of the eggs during the storage in water. Final HR also includes the hatch rate of the eggs at the end of the period of water storage, after immersion in a bacterial broth solution. R indicates the Pearson correlation coefficient between hatch rate and storage duration, and P the significance of the correlation (*P<0.05, **P<0.001 and ***P<0.0001)

## Discussion

*Aedes albopictus* and *Ae. aegypti* eggs hatch efficiently in a solution of bacterial broth while there is lower hatching in deionized water. Fallis and Slow [16] observed such differences in *Ae. punctor* eggs, with no eggs hatching in deionized water alone and a variable rate between 30 and 90 % with the addition of bacterial broth. The fact that the bacterial broth hatching medium is efficient in inducing hatching of both *Ae. albopictus* and *Ae. aegypti* is interesting. Indeed, it is known that hatching can be induced by a different concentration of dissolved oxygen depending on the species, as observed by Judson al. [25] for *Ae. sierrensis* and *Ae. aegypti*. Moreover, Schwan and Anderson [18] also observed variation in dissolved oxygen concentration required for optimal hatching of different strains of *Ae. sierrensis*. The high efficiency of our hatching medium could be due to the fact that both species might require the same dissolved oxygen concentration to hatch, or because the bacterial broth caused a progressive reduction in dissolved oxygen concentration with time, inducing both species to hatch. In a situation of mass production of both species in the same facilities, this common hatching medium may reduce costs and simplify the operation.

The use of boiled water induced the hatching of few eggs in *Ae. albopictus* while it induced a higher proportion of *Ae. aegypti* eggs to hatch, though the highest hatch rate was induced by submergence in bacterial broth solution. Boiling is known to remove all the oxygen from water, whereas bacterial metabolism in a broth solution deoxygenates water over time. This complete deoxygenation of water by boiling prior to egg submersion seemed to favour hatching of *Ae. aegypti* more than *Ae. albopictus*. However, for *Ae. aegypti*, when the duration of storage increased, the hatch rate decreased. In *Ae. aegypti*, fresh eggs have been seen to hatch similarly in dechlorinated water and in rearing medium [26], but water alone is not sufficient to allow hatching of eggs that have been stored for longer than two weeks. This is probably due to the fact that, over time, *Ae. aegypti* embryos enter dormancy, at the end of embryogenesis [27–29]. For *Ae. albopictus*, the entrance into dormancy is probably very fast, just after the drying process, since whatever the storage duration few eggs hatched in water. As soon as the eggs enter dormancy, water alone is probably not a favourable enough medium for larval development while the lower oxygen content of bacterial broth solution or boiled water are suitable to interrupt dormancy [27, 29]. As soon as those eggs, first put in boiled deionized water, are placed in bacterial broth solution, most of them hatch, up to the same rate as if they were put first in the bacterial broth. Thus the use of boiled water as a low cost solution for egg hatching is not viable for mass rearing of either *Ae. aegypti* or particularly *Ae. albopictus*. Physical hatching stimuli were not explicitly considered in this study, though efforts were made to maintain a constant environment and treat all egg batches in the same manner. But, for example, relative agitation of the water may be important in some species [30], though Borg and Horsfall [14] showed chemical stimuli to be stronger than physical ones in *Ae. aegypti*.

In our storage conditions, the quality of the eggs appeared to be good (a hatch rate of more than 80 %) for the first 10 weeks, after which quality decreased to reach a null hatch rate after 4 months. Previous studies on *Ae. aegypti* have shown similar patterns of egg quality decline with storage duration. High relative humidity (RH) allows the storage of eggs without loss of egg viability [28, 31, 32]. After the pre-hatch conditioning, eggs should be stored in sufficiently high RH to avoid egg desiccation but sufficiently low to avoid hatching. Morlan et al. [20] stored *Ae. aegypti* eggs in a covered glass cup for 10 weeks and achieved a hatch rate varying between 78 and 92 %, while Ansari et al. [26] observed 73 % hatching after 12 weeks from eggs stored in polythene bags at 29 °C and 80–85 % RH. *Aedes* eggs have also been stored in a plastic container with a saturated solution of potassium chlorate to maintain humidity at an optimal 85 % [33].

The decrease in quality could be due to a lethal loss of water; a strong correlation between changes in weight and hatch rate has been already demonstrated [28, 34]. In our experiment the same correlation was seen: the number of collapsed eggs increased with storage duration.

We have demonstrated that storage in water is an alternative solution for efficient egg storage which retains viability for longer periods than conventional storage. Indeed, for both *Aedes* species tested, after 5 months of storage a hatch rate of 85 % was observed for both species with no impact of storage duration on *Ae. albopictus* hatch rate though a significant decrease in hatch rate with time was observed for *Ae. aegypti*. Moreover, for both species, a significant increase in the number of eggs hatching in the water was observed with duration of storage, as observed by Fallis and Slow for *Ae. punctor* [16], probably because of the natural loss of oxygen in the water with time, that could be accelerated by the decomposition of the dead bodies of hatching larvae and the resulting bacterial growth. Oxygenation of the water by bubbling oxygen through the storage tray could probably prevent this phenomenon.

An alternative reading of this delayed hatching in some eggs is the phenomenon of scattered, or installment, hatching, as first described to our knowledge by Gillett [35]. Paired with the skip oviposition employed by *Aedes* females who distribute their egg batches between a number of oviposition sites even within the same gonotrophic cycle, installment hatching is caused by a variety in depth of diapause between individual eggs. Gillett at first failed to identify any external factor which could explain the observed variety of inter- or intra-egg batch response to hatching stimuli, and postulated that an inherited factor was somehow responsible. However, later research demonstrated that the higher the number of bacteria populating the surface of an egg, the smaller the stimuli required to induce hatching, and that clumped eggs are more likely to hatch than more evenly distributed eggs due to the bacterial density [36]. It was even shown that the larvae hatching from the first eggs to hatch feeding on the bacteria on neighbouring eggs is enough to delay their hatching. The result has subsequently been reproduced, though the mechanism is still not clear [37]. This phenomena may have an impact on the optimal way to store eggs; prior surface sterilization may reduce hatching during water storage, as might decreasing the density of eggs in the water, distributing or agitating them in some way.

In *Aedes* mass rearing facilities, management of eggs is essential to allow the efficient production of millions of sterile males. After the collection of eggs from mass rearing cages [12], they can be stored at 27 ± 1 °C for 10 weeks without any loss of quality. If a longer period of storage is needed, another option is to store egg-papers in oxygenated-water. After egg storage, the best method of hatching for both *Ae. aegypti* and *Ae. albopictus*, regardless of the duration of storage, is submersion in a bacterial broth solution. After hatching, larvae need then to be distributed into a larval rearing mass device that could be the mechanized stainless steel rack holding 50 mass-rearing trays developed by the Joint FAO/IAEA IPCL [13]. Further studies are now needed to improve the development of larvae in this rack, the collection of the pupae and the management of mass rearing adult cages stocked with those pupae.

## Conclusion

The methods for management of eggs from two key vector mosquito species, *Aedes aegypti* and *Ae. albopictus*, were investigated in conditions of large scale laboratory rearing. The ability to store eggs over time and stockpile in preparation for colony upscaling or production of material for release will be key in the management of mass rearing facilities of these species, a crucial step in the application of the sterile insect technique which is being trialled as a population control method in various countries. The majority of eggs stored in conditions of high humidity and temperatures used to rear mosquitoes hatched for the first 10 weeks of storage, after which hatch rate declined, whereas a novel method of storing eggs in water extended this period up to 5 months. This development will greatly increase the flexibility available in managing *Aedes* colonies; further improvement to prevent the low level of hatching over time will enhance this benefit. Both species were shown to hatch most efficiently when submerged in a bacterial broth solution, and the availability of a method of hatching which is common to both species will provide operational simplicity in facilities where both vectors of diseases such as dengue and chikungunya are being reared for suppression programmes.
